# Tensiomyography Derived Parameters Reflect Skeletal Muscle Architectural Adaptations Following 6-Weeks of Lower Body Resistance Training

**DOI:** 10.3389/fphys.2019.01493

**Published:** 2019-12-10

**Authors:** Matthew T. Wilson, Andrew M. F. Ryan, Scott R. Vallance, Alastair Dias-Dougan, James H. Dugdale, Angus M. Hunter, D. Lee Hamilton, Lewis J. Macgregor

**Affiliations:** ^1^Physical Exercise and Nutrition Research Group, Faculty of Health Sciences and Sport, University of Stirling, Stirling, United Kingdom; ^2^School of Exercise and Nutrition Sciences, Institute for Physical Activity and Nutrition, Deakin University, Geelong, VIC, Australia

**Keywords:** muscle architecture, pennation angle, resistance training, tensiomyography, skeletal muscle hypertrophy

## Abstract

Measurement of muscle specific contractile properties in response to resistance training (RT) can provide practitioners valuable information regarding physiological status of individuals. Field based measurements of such contractile properties within specific muscle groups, could be beneficial when monitoring efficacy of training or rehabilitation interventions. Tensiomyography (TMG) quantifies contractile properties of individual muscles via an electrically stimulated twitch contraction and may serve as a viable option in the aforementioned applications. Thus, aims of this study were; (i) to investigate the potential use of TMG to quantify training adaptations and differences, in response to exercise specific lower limb RT; and (ii) investigate any associations between TMG parameters and accompanying muscle architectural measures. Non-resistance trained male participants (*n* = 33) were randomly assigned to 1 of 3 single-exercise intervention groups (*n* = 11 per group); back squat (BS), deadlift (DL), or hip thrust (HT). Participants completed a 6-week linearized training program (2× per week), where the assigned exercise was the sole method of lower body training. Pre- and post-intervention testing of maximal dynamic strength was assessed by one repetition maximum (1RM) of BS, DL, and HT. Radial muscle belly displacement (Dm) and contraction time (Tc) were obtained via TMG from the rectus femoris (RF) and vastus lateralis (VL) pre- and post-intervention, alongside muscle architectural measures (pennation angle and muscle thickness). All three groups displayed significant increases all 1RM strength tests (*p* < 0.001; pη2 = 0.677–0.753). Strength increases were accompanied by significant overall increases in RF muscle thickness (*p* < 0.001, pη2 = 0.969), and pennation angle (*p* = 0.007, pη2 = 0.220). Additionally, an overall reduction in RF Dm (*p* < 0.001, pη2 = 0.427) was observed. Significant negative relationships were observed between RF Dm and pennation angle (*p* = 0.003, *r* = −0.36), and with RF Dm and muscle thickness (*p* < 0.001, *r* = −0.50). These findings indicate that TMG is able to detect improved contractile properties, alongside improvements in muscle function within an untrained population. Furthermore, the observed associations between Dm and muscle architecture suggest that TMG contractile property assessments could be used to obtain information on muscle geometry.

## Introduction

Resistance training (RT) is a key component within most athletes’ training routines (Parsons, 2010; García-Pallarés and Izquierdo, 2011) and if carried out at appropriate frequency and duration, RT increases muscular mass and strength (Schoenfeld et al., 2016). Furthermore, strength gains attained from appropriate strength training often transfer to improved sporting performance measures such as jump height (Fitzpatrick et al., 2019) and sprint performance (Harries et al., 2018). In order to maximize transfer of strength to performance, closed-chain RT exercises involving similar movement patterns to sporting performance are recommended (Seitz et al., 2014; Burnie et al., 2017). As such, knee extensors are a common focus of RT owing to their involvement in athletic movement patterns (e.g., sprinting and jumping) (Jacobs et al., 1996; Simsek et al., 2016; Howard et al., 2017). Commonly, the efficacy of such RT is commonly measured by 1 maximal repetition (1RM) tests in strength and conditioning settings (Levinger et al., 2009), providing an indication of any change observed in muscle function. Externally loaded free-weight back squats (BSs) are a popular exercise held in high regard in RT and rehabilitation. The BS’s ability to activate the full closed kinetic chain, from requirement of torque being produced through multiple joints in the body; whilst maintaining a fixed foot position (Clark et al., 2012; Kwon et al., 2013). Performing BSs has been shown to recruit knee extensor musculature as prime movers alongside the hip, with trunk and back musculature acting in a stability/postural capacity (Caterisano et al., 2002; Schoenfeld, 2010; Marchetti et al., 2016). Conventional deadlifting is also commonly used in lower body RT. Not only does the deadlift (DL) elicit high activation of knee musculature, but also shares biomechanic and muscle activation similarities around the hip and knee joints with sport specific movement patterns such as vertical jumping (Escamilla et al., 2002). Additionally, the hip thrust (HT) has seen increasing prevalence in RT due to its versatility in increasing lower limb strength within different training scenarios (Collazo García et al., 2018). Electromyography profile comparisons have shown the HT able to elicit comparative knee extensor, knee flexor and hip extensor activation with the BS (Contreras et al., 2015) and the DL (Andersen et al., 2018). In particular Contreras et al. (2015) showed statistically similar levels of VL peak activation between the HT and BS. It is on the basis of muscle activation profiles that the HT training has caused improvements in sporting performance measures (Contreras et al., 2017), and improved muscle function in clinical rehabilitation (Vinstrup et al., 2017). Furthermore the unique ROM and load positioning of the HT support its use as alternative to other closed-chain, lower limb resistance exercises whilst still providing sufficient loading stimuli and muscle activation required to causes adaptations (Neto et al., 2019). However, whilst training efficacy of the BS, DL, and HT has been demonstrated, showing increases in knee extensor strength and force production (Thompson et al., 2015; Fitzpatrick et al., 2019), the underpinning physiological adaptations that are responsible are not fully understood. By investigating these adaptive responses within specific muscle groups such as the quadriceps, greater clarity can be provided to coaches and practitioners on the efficacy of commonly employed resistance exercises for performance and rehabilitation settings.

Strength gains made through RT have been observed alongside alterations within muscle morphology and architecture (Reeves et al., 2004; Alegre et al., 2006; Franchi et al., 2014; Kim et al., 2015; Nóbrega et al., 2018). Muscles with fibers running parallel to tendons are said to have a longitudinal architectural arrangement, which differs from muscles in which fibers orientate at an angle to the direction of force generation (unipennate), or at more than one angle (multi-pennate) (Lieber and Fridén, 2000). Unipennate arrangement of muscles such as vastus lateralis (VL), and bipennate arrangement of muscles such as rectus femoris (RF) are better suited to produce higher forces than longitudinal muscle heads (Blazevich, 2006). Following periods of between 5 and 14 weeks, increases in architectural elements such as muscle thickness and angle of fiber pennation (pennation angle) are associated with improvements in force production (Aagaard et al., 2001; Blazevich et al., 2003). Associations between increased pennation angle and force production seen following RT are thought to be due to increases in physiological cross sectional area (PCSA) (Seynnes et al., 2007; Campbell et al., 2013; Vieira et al., 2018). With PCSA being the sum of the CSA of all fascicles within the muscle, increases in pennation angle (up to 45°) results in greater transmission of force to direction of pull [($\frac{1}{1}\,S\,i\,n\,\left(2\,{\text{θ}}_{\text{p}}\right)$; where θ_p_ is pennation angle] (Alexander and Vernon, 1975; Rutherford and Jones, 1992). Furthermore, increased pennation angle also leads to increased muscle tetanic tension, to which PCSA is directly proportional (Lieber and Fridén, 2000). Such alterations in muscle architecture following RT could affect contractile properties of muscles, which may be assessed using objective, non-invasive mechanomyographic methods such as tensiomyography (TMG).

Tensiomyography assesses contractile properties of an isolated muscle by measuring a number of parameters in response to a twitch contraction (Valenčič and Knez, 1997). Such parameters, including contraction time (Tc) and radial muscle belly displacement (Dm), can be obtained quickly and with minimal input from the participant being assessed. Tc has been previously correlated with proportions of slow twitch fibers within lower limb muscles, providing construct validation for TMG (Valencic et al., 2001; Dahmane et al., 2006; Šimunic et al., 2011); whilst a shorter Tc being considered reflective of a greater rate of force production (Rusu et al., 2013). Within the literature, Dm is considered to reflect muscle belly stiffness (Whitehead et al., 2001) and has been shown to alter with changes in muscle fatigue and aging (Rusu et al., 2013; Macgregor et al., 2016). TMG can distinguish between muscles of different training status, with shorter Tc and smaller Dm being seen in athletes with greater exposure to strength and power training (Loturco et al., 2015; De Paula Simola et al., 2016; Šimunič et al., 2018); due to increased proportions of fast twitch muscle fibers, and greater amounts of contractile material, respectively. Additionally, atrophy induced changes in muscle architecture are associated with increased Dm (Pišot et al., 2008; Šimunič et al., 2019). Whilst the direct purposes of the aforementioned studies was to apply TMG in longitudinal and clinical settings, the resulting data does provide a level of construct validation for Dm alongside measures of muscle architecture. From these data, it is conceivable that the association between Dm and muscle architecture could be reflected following changes in muscle hypertrophy.

However, whilst both Tc and Dm have shown good-excellent inter-rater and inter-day reliability (Križaj et al., 2008; Tous-Fajardo et al., 2010; Šimunič, 2012), a recent review by Macgregor et al. (2018) highlighted scarcity of data surrounding use of TMG in a longitudinal context, particularly concerning training interventions. Beyond the above-mentioned atrophy studies, only one study has investigated long term stability of TMG (Ditroilo et al., 2013), showing good absolute and relative long term reliability for Tc and Dm. Whilst using twitch torque assessment methods could be of use in monitoring rehabilitation interventions, Macgregor et al. (2018) also noted the requirement to integrate TMG parameters of contractile properties, namely Dm, alongside established physiological measures to strengthen its validation in longitudinal contexts. Therefore, as a proof of principle for the use of TMG to monitor muscular adaptations, we designed this study to assess the TMG responses within knee extensor musculature alongside alterations in muscle architecture following 6 weeks of RT utilizing common lower limb resistance exercises.

The aims of this study were: (1) to investigate the potential use of non-invasive contractile property assessments to quantify training adaptations, in response to 6 weeks of exercise specific lower limb RT and; (2) to investigate any association between contractile parameters and accompanying muscle architectural changes within the knee extensors.

We hypothesized (1) that knee extensor Dm reductions would be observed following RT, alongside increases in muscle thickness and pennation angle. From previous literature it was hypothesized (2) that these responses would be larger following BS training, compared to the other exercises. Furthermore, we hypothesized (3) that muscle belly Dm obtained through TMG would be associated with ultrasonography measures of pennation angle and muscle thickness; thus, providing evidence to support validity of TMG’s longitudinal application alongside established physiological markers.

## Materials and Methods

### Participants

Eligible participants were physically active, but non-resistance trained males (recreational sports participants with >3 h/week of self-reported physical activity). To control for differing muscle strength and hypertrophy responses across different phases of the menstrual cycle (Sung et al., 2014) women were excluded from the study. All participants were required to maintain their normal exercise routines throughout the study (primarily consisting of non-contact team sports, running, cycling and swimming), to be free of musculo-skeletal injury for the previous 2 years and to complete a physical activity readiness questionnaire prior to beginning the study. An *a priori* power analysis was conducted for increases in 1RM BS strength (as a measure of dynamic muscle strength and function) [α = 0.05; β = 0.8; Effect size: 0.4 (Zweifel et al., 2017)]. It was determined a total sample of 21 participants (9 per group) was required to achieve statistical power of 0.86 for changes in 1RM BS strength (G^∗^Power 3.1). To maximize statistical power and account for potential dropouts, a convenience sample of 48 voluntary participants (16 per group) was recruited for the study. Of the 48 recruited participants, 10 withdrew from the study due to injuries sustained in team sports, 3 were excluded for failing to complete the training intervention (<90% attendance), and 2 were unable to complete post-intervention testing, leaving a sample of 33 participants (height; 181.15 ± 6.15 cm, body mass; 79.32 ± 11.96 kg). Participants were randomly assigned to one of three training groups by computer generated numerical coding (Schulz et al., 2010); squat group (*n* = 11): 179.33 ± 5.99 cm, 79.02 ± 17.91 kg, DL group (*n* = 11): 180.27 ± 6.66 cm, 78.29 ± 6.97 kg, HT group (*n* = 11): 182.78 ± 5.75 cm, 81.04 ± 11.53 kg. This study received institutional ethical approval from the University of Stirling’s School of Sport Research Ethics Committee and was conducted in accordance with the Declaration of Helsinki.

### Experimental Design

**FIGURE 1:**
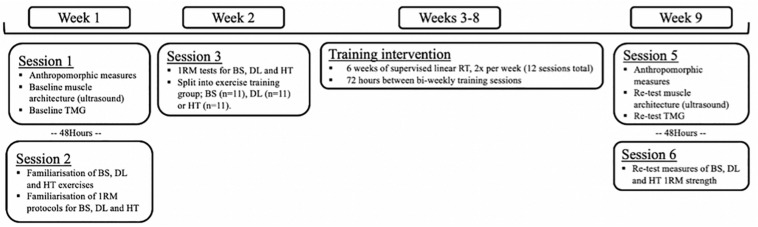
Schematic timeline of experimental design. TMG, tensiomyography; BS, back squat; DL, deadlift; HT, hip thrust; 1RM, one maximal repetition; RT, resistance training.

A three-group parallel, repeated measures design was implemented to investigate contractile properties and muscle architecture adaptions in response to three different strength training exercise programs (Figure 1). In week 1, participants were familiarized with all testing procedures across two laboratory sessions and divided into three training groups. Session 1 included anthropomorphic measures, muscle architecture assessment and TMG contractile property assessment. These data were used as the non-resistance trained baseline measures, to avoid the confounding effects of the following strength assessments and were all recorded from the participants dominant limb. Session 2 included familiarization and initial performance of 1RM procedures for the BS, DL, and HT exercises (Logan et al., 2000). In week 2, only the 1RM session was repeated to obtain baseline strength measures, which was structured by 1RM data obtained during familiarization to control for fatigue. Participants were then informed of their allocated training groups and were familiarized with their respective exercise-specific training program. Participants then completed a 6-week program with two sessions a week, and each session being completed with coaches present to ensure adherence and correct technique. Upon completion of the supervised 6-week program (2x sessions per week, separated by 72 h), participants completed identical testing procedures to week 1.

### Ultrasound Assessment

**FIGURE 2:**
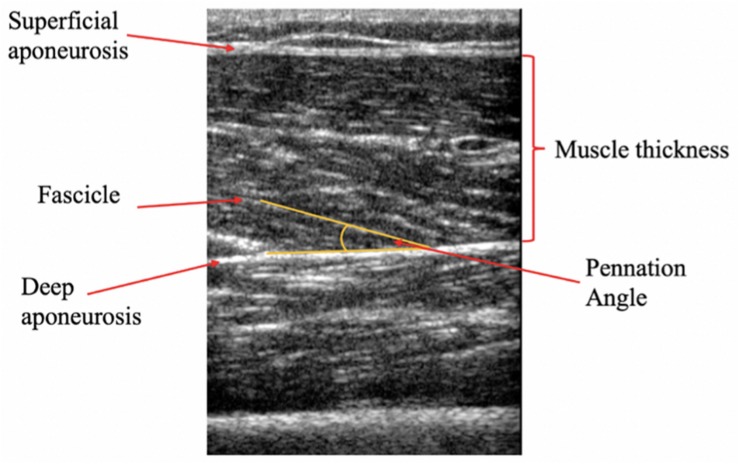
Representative longitudinal B-mode ultrasound image of the Vastus lateralis (VL). Muscle pennation angle is represented at the angle between the fascicle and the deep aponeurosis, whilst muscle thickness is defined as the distance between the inside borders of the deep and superficial aponeurosis.

Upon arrival participants were asked to rest quietly for 10 min on a bed, in a supine position to account for any redistribution of body fluids. Participants remained in the supine position for the duration of the Ultrasound examination. This position was maintained for TMG assessment (with addition of the custom angled pillow under the investigated leg). The measurement sites of the VL and RF muscles were marked and recorded according to anatomical landmarks used in the literature (Blazevich, 2006). Briefly, images of VL were taken at 36% (distal) of the distance between the superior border of the patella and the anterior superior iliac spin. Images of the RF were taken at 57% of the distance between the superior edge of the patella and the anterior superior iliac spine. The RF measurement site was chosen as the mid-point of the muscle belly, previously showing higher CSA values compared to the distal region (Matta et al., 2014). This region was also selected due to the measurement site being the same to that of TMG transducer probe; the authors considered it more appropriate to associate measures of muscle architecture from this region with TMG parameters obtained from the RF muscle belly. These sites were then replicated post-intervention. Two-dimensional brightness-mode ultrasound images were taken for each muscle using an HDI-5000 scanner (ATL Ultrasound, Bothell, WA, United States) with a 7- to 12-MHz linear transducer 5 cm probe. A water-soluble ultrasound gel (Healthlife, Barclay-Swann Ltd., United Kingdom) was used to ensure optimal image quality whilst minimizing pressure upon the participant’s skin. Pennation angle was identified as the angle between a muscle fascicle and its deep aponeurosis (Blazevich et al., 2006; Figure 2). Muscle thickness was identified as the distance between the deep and superficial aponeurosis at the ends of each image (Blazevich et al., 2006; Figure 2). Three images were taken at each site in a longitudinal plane, and then exported to Image J (National Institutes of health, Bethesda, MD, United States, version 1.8.0_112) for analysis. The average value from the three images were used for analysis (Aagaard et al., 2001); a methodology that has shown high absolute and relative reliability for architectural parameters being measured in this way (Ema et al., 2013b; König et al., 2014; Silva et al., 2018). All images were taken from the participants’ dominant limb (defined as the leg with which they would kick a ball) by the same experienced sonographer. Repeated measurement intraclass coefficient of variation (ICC) and coefficient of variation (CV) were previously established in a pilot study for: muscle thickness (RF; CV: 2.86%, ICC: 0.97, VL; CV: 4.52%, ICC: 0.89) and pennation angle: (RF; CV: 5.38%, ICC: 0.94, VL; CV: 5.67%, ICC: 0.94).

### Tensiomyography Assessment

**FIGURE 3:**
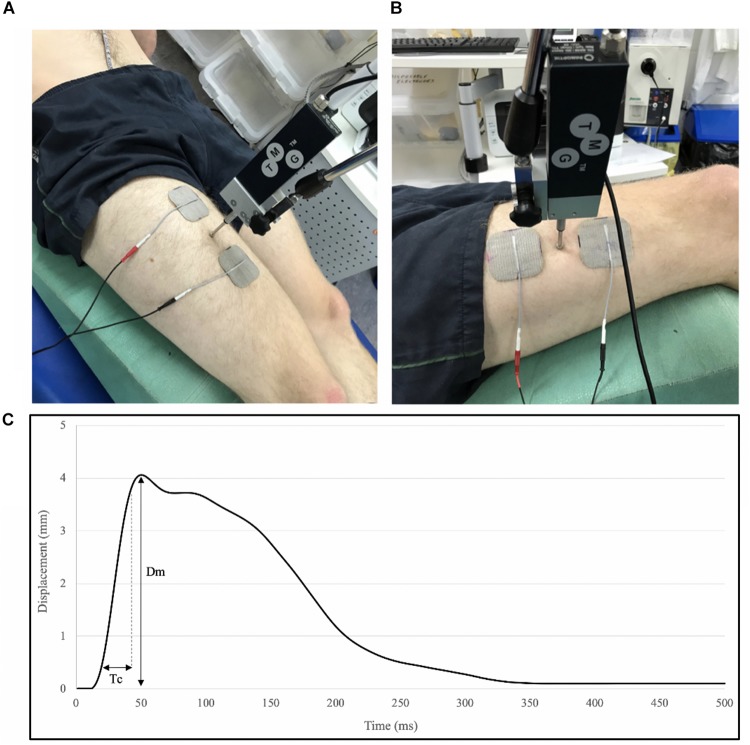
Tensiomyography set-up for measurement of the Rectus femoris (RF) **(A)** and the VL **(B)**, and parameters extracted from a typical TMG trace; displacement (Dm) and contraction time (Tc) between 10% and 90% of Dm **(C)**. Electrode and stimulator position were recorded with reference to anatomical landmarks and replicated in post-intervention measures.

Tensiomyography assessment of the RF and VL were carried out immediately following the ultrasound assessment. Participants remained in a supine position with a supportive pad placed underneath the knee of the dominant leg, to maintain 60° knee angle (0° = full knee extension) throughout the assessment. Hip angle was maintained and controlled with angles of hip flexion ranging from 33° to 40° (0° = full hip extension) due to anatomical differences within the study’s cohort. Participant positions were replicated for post-intervention testing. The measurement sites of the VL and RF were identified using manual palpation to locate the thickest part of the muscle belly (Ditroilo et al., 2013). A digital TMG sensor (GK 40, Panoptik d.o.o., Ljubljana, Slovenia) was placed perpendicular to the skin surface upon the point of maximal muscle belly Dm. Two self-adhesive surface electrodes (5 cm^2^) (Axelgaard, United States) were placed on either side of the sensor (5 cm from the midpoint of the electrode), whilst ensuring not to cross the muscle borders so as to avoid co-activation (Figures 3A,B). The position of electrodes and transducer probe were marked and measured with reference to anatomical landmarks (anterior iliac crest and superior border of the patella) and recorded for replication post-intervention. A single 1 ms wide stimulation was applied at the initial intensity of 20 (milliamperes) mA at a constant of 30 V, with the progressive increase in amplitude of subsequent stimulations, until the maximal Dm of the muscle, measured by the linear transducer, was achieved (Figure 3C). An inter-stimulation time interval of 10–15 s was used. The parameters of Dm [maximal radial Dm of the twitch contraction (mm)] and Tc [time between 10% and 90% of Dm (ms)] were extracted from the maximal twitch response of the muscle, by TMG software (Version 3.6.16) and used for offline analysis.

### 1RM Testing for the Back Squat, Deadlift, and Hip Thrust

Participants performed 1RM protocols in BS, HT, and DL in each session, the order of exercises was randomized. All participants used the same barbell and weight plates (Eleiko, Sweden), and in a FT700 power rack (Fitness technologies, Australia) with safety bars and with trained spotters present. In addition, a bar pad and wrist straps (Gunsmith fitness, United Kingdom) were used for the HT and DL exercises, respectively. In week 1 participants received formal introduction and coaching on correct technique to each exercise by a qualified strength and conditioning coach (see Supplementary Material), after completing a standardized warm-up of static and dynamic stretching. National Strength and Conditioning Association (NSCA) guidelines for exercise technique were used to coach the BS, DL, and HT (Hales, 2010; Contreras et al., 2011; Comfort et al., 2018). To ensure minimal fatigue, the p1RMs achieved in familiarization were used to structure 1RM testing the following week (baseline measurement), which followed the same sets and percentage increments. Once the participants felt comfortable and confident in their technique, they completed a 1RM testing protocol for each exercise (Logan et al., 2000). Briefly, this required participants to perform 5 repetitions (reps) at 50% of their individual predicted 1RM (p1RM), 3 reps at 75% p1RM, 3 further reps at 85% p1RM and 2 reps at 90% p1RM. Participants rested 2–4 min between each set. Participants then attempted 1 rep at 100% p1RM. If the attempt was successful a 5% increase of p1RM was added, and another attempt was performed (105%p1RM) following a rest of 2–4 min. In the event of a failed 1RM attempt, the participant rested 2–4 min and then re-attempted the same load. If the second 1RM attempt was also failed participants performed no further 1RM attempts, and the last successfully lifted load was taken as 1RM. In all 1RM testing, a rating of perceived exertion (RPE) scale was used for participant safety as well as guiding incremental increases in the weight lifted (0 meaning no effort, and 10 meaning maximal effort/volitional fatigue) (Zourdos et al., 2016). Specifically, when a participant’s RPE reached 9/10, 1RM attempts were recorded. Individual participant stance width, foot angle, and distance from feet to box (HT only), were recorded in accordance with each exercise’s technique guidelines and marked each time for replication (Schoenfeld, 2010).

### Training Intervention

**FIGURE 4:**
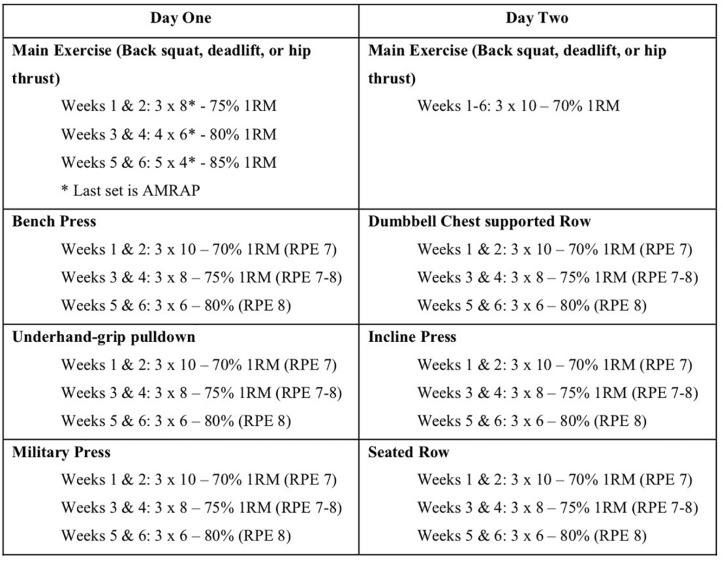
Linearized training protocol used for the 6-week intervention (weeks X, number of sets × number of reps). Participants had a 72-h break between training sessions, which were conducted at the same time each day, and provided with 40 g of whey protein supplementation. All other exercises were performed in seated position to reduce the involvement of the lower limbs (RT, resistance training; AMRAP, as many reps as possible before failure or form was compromised; RPE scale used during sessions as a training intensity guide).

Participants attended two supervised training sessions per week (approximately 72 h apart) for 6 weeks. Each group used only their respective exercise as the method of lower body exercise, whilst the rest of the program remained consistent for all groups (Figure 4). All loads for the respective exercise sets were calculated according to the participant’s previously achieved 1RM value. The program followed a linearized progression model to ensure a sufficient intensity within each session (Fleck, 1999). A set of as many repetitions as possible (AMRAP) was included at the end of session 1 each week, to try and maximize the potential for a training response (Morton et al., 2016). All additional exercise loads were calculated according to a combination of estimated 1RM scores, and the RPE scale. The coaches present at each session ensured that participants maintained correct technique for each exercise, and spotted participants when the exercise required. Following the completion of each training session (and all 1RM testing sessions), participants were provided with 40 g of whey protein in a drink to aid in muscle recovery and muscle building (Macnaughton et al., 2016).

### Statistical Analysis

All statistical analysis was carried out on Graphpad, Prism (Graphpad Software, CA, United States). Baseline and post-intervention scores for ultrasound, TMG and 1RM assessments were assessed for normality (Shapiro-Wilk test). Two-way repeated measure ANOVAs were used to determine the main effects of the three training interventions upon measures of 1RM strength, muscle architecture and the TMG parameters of each assessed muscle [three training groups x two time-points (pre-and post-training)], individually. Pearson’s product-moment correlation coefficient was used to investigate any potential relationships between muscle architecture and contractile properties within the RF and VL. Correlation coefficients of 0.1–0.3, 0.31–0.5, 0.51–0.7, and >0.71 were classified as small, moderate, large and very large correlations, respectively (Cohen, 1988). Statistical significance was set at *p* < 0.05 (^∗^). All data are reported as mean ± standard deviation, and with changes represented as percentage ± upper and lower 95% confidence intervals (CI). Partial eta squared (pη2) effect sizes were calculated, with 0.01, 0.06, and 0.14 considered small, medium and large, respectively (Cohen, 1988).

## Results

Of the 48 participants recruited, 33 completed the 6 weeks of exercise specific lower body training and all assessments, with an average training attendance of 95.7%. All 3 exercise groups displayed significant increases over time in each of the three 1RM tests (Table 1); BS [*F*_(__1_,_30__)_ = 91.41, *p* < 0.001, pη2 = 0.753]; DL [*F*_(__1_,_30__)_ = 63.00, *p* < 0.001, pη2 = 0.677]; HT [*F*_(__1_,_30__)_ = 78.13, *p* < 0.001, pη2 = 0.723]. However, no interaction was found between the exercise groups for each of the three exercises; BS [*F*_(__2_,_30__)_ = 3.276, *p* = 0.052, pη2 = 0.179]; DL [*F*_(__2_,_30__)_ = 1.463 *p* = 0.248, pη2 = 0.089]; HT [*F*_(__2_,_30__)_ = 0.601, *p* = 0.056, pη2 = 0.039].

**TABLE 1:** Pre, Post, and% changes of 1 rep max strength in response to 6 weeks of exercise specific lower body training, and baseline anthropometrics.

	**Squat group**			**Deadlift group**			**Hip thrust group**		
**Measure**	**Pre**	**Post**	**Δ% (CI)**	**Pre**	**Post**	**Δ% (CI)**	**Pre**	**Post**	**Δ% (CI)**
Back squat 1RM (kg)	91.8 ± 20.9	104.2^∗^±21.9	14.0(9.3,18.7)	87.4 ± 16.9	95.0^∗^±13.9	9.9(4.1,15.8)	91.3 ± 14.7	98.2^∗^±12.1	8.3(4.2,12.3)
Deadlift 1RM (kg)	117.0 ± 23.1	127.5^∗^±19.4	10.3(3.0,17.5)	111.8 ± 21.4	129.6^∗^±21.7	16.76(9.1,24.4)	114.2 ± 16.6	127.8^∗^±18.4	12.2(7.7,16.7)
Hip thrust 1RM (kg)	134.7 ± 27.4	156.7^∗^±31.8	16.6(10.8,22.5)	136.6 ± 24.6	155.2^∗^±30.9	13.72(6.3,21.1)	140.1 ± 20.02	165.4^∗^±22.8	18.6(11.3,25.9)
Height (m)	179.3 ± 6.0			180.2 ± 6.7			182.8 ± 5.8		
Body mass (kg)	79.0 ± 17.9			78.3 ± 6.9			81.0 ± 11.5		

*All data presented as mean ± SD; CI, 95% confidence limits; ^∗^, significant increase over time, *P* < 0.05.*

**FIGURE 5:**
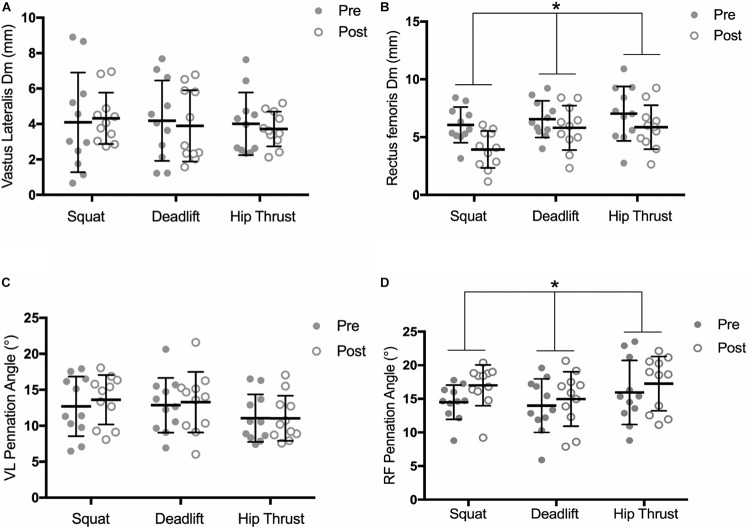
Pre and post training intervention measures of; radial muscle Dm in the VL **(A)** and RF **(B)**, and pennation angle of the VL **(C)** and RF **(D)**, by exercise training group. Individual responses are shown with gray and white circles. Error bars display the means ± SD. ^∗^, significant time effect observed (ANOVA, *P* < 0.05).

There was no interaction observed for pre-post Dm measures of the RF between the 3 exercise groups, over time [*F*_(__2_,_30__)_ = 2.041, *p* = 0.148, pη2 = 0.114]. However, a significant time effect was observed across the three exercise groups, [*F*_(__1_,_30__)_ = 22.37, *p* < 0.001, pη2 = 0.427]. Analysis of the pre-post Dm measures of the VL demonstrated no significant interaction [*F*_(__2_,_30__)_ = 0.289, *p* = 0.751, pη2 = 0.019], or time effect [*F*_(__1_,_30__)_ = 0.141, *p* = 0.710, pη2 = 0.005] (Figures 5A,B). No time effects were observed for Tc changes in either the VL [*F*_(__1_,_30__)_ = 0.7112, *p* = 0.406, pη2 = 0.023] or the RF [*F*_(__1_,_30__)_ = 0.028, *p* = 0.869, pη2 = 0.0009]. Similarly, no interactions were observed between exercise groups for Tc changes, in either the VL [*F*_(__2_,_30__)_ = 0.336, *p* = 0.718, pη2 = 0.022] or the RF [*F*_(__2_,_30__)_ = 0.651, *p* = 0.529, pη2 = 0.042].

Significant time effects were observed for muscle thickness changes in both the RF [*F*_(__1_,_30__)_ = 30.9, *p* < 0.001, pη2 = 0.969] and the VL [*F*_(__1_,_30__)_ = 33.02, *p* < 0.001, pη2 = 0.524]. However, no interactions were observed between exercise groups in the RF [*F*_(__2_,_30__)_ = 1.576, *p* = 0.223, pη2 = 0.612], or the VL [*F*_(__2_,_30__)_ = 0.795, *p* = 0.461, pη2 = 0.050] (Table 2). No interaction was found for RF pennation angle changes between the exercise groups over time [*F*_(__2_,_30__)_ = 0.719, *p* = 0.50, pη2 = 0.045], however, a significant time effect was observed across the 3 groups, [*F*_(__1_,_30__)_ = 8.459, *p* = 0.007, pη2 = 0.220] (Figures 5C,D). Analysis of pre-post change in VL pennation angle displayed no significant interaction [*F*_(__2_,_30__)_ = 1.126, *p* = 0.338, pη2 = 0.070], or time effect [*F*_(__2_,_30__)_ = 2.989, *p* = 0.094, pη2 = 0.091].

**TABLE 2:** Pre, Post, and changes of muscle architecture and contractile properties of quadriceps muscles, in response to 6 weeks of exercise specific lower body training.

		**Squat group**			**Deadlift group**			**Hip thrust group**		
**Muscle**	**Measure**	**Pre**	**Post**	**Δ% (CI)**	**Pre**	**Post**	**Δ% (CI)**	**Pre**	**Post**	**Δ% (CI)**
Vastus lateralis	Pennation angle (°)	12.7 ± 4.1	13.6 ± 3.4	0.9(−0.2,2.1)	12.6 ± 3.8	13.3 ± 4.2	0.4(−0.7,1.6)	11.1 ± 3.31	11.0 ± 3.14	−0.1(−1.15,1.11)
	Muscle thickness (cm)	1.9 ± 0.5	2.1^∗^±0.5	0.2(0.1,0.3)	2.0 ± 0.5	2.2^∗^±0.5	0.2(0.1,0.3)	2.0 ± 0.48	2.1^∗^±0.53	0.1(−0.02,0.3)
	Dm (mm)	4.1 ± 2.8	4.2 ± 1.5	0.2(−1.4,1.8)	4.2 ± 2.3	3.9 ± 2.0	−0.3(−1.1,0.4)	4.0 ± 1.8	3.7 ± 1.0	−0.3(−1.5,1.0)
	Tc (ms)	22.0 ± 6.2	25.1 ± 2.9	3.1(−0.5,6.5)	22.00 ± 5.9	22.7 ± 4.0	0.7(−2.8,4.2)	24.7 ± 5.9	27.2 ± 5.5	2.5(−2.1,7.0)
Rectus femoris	Pennation angle (°)	14.5 ± 2.6	17.0^∗^±3.1	2.5(0.1,4.9)	14.0 ± 3.9	14.9^∗^±4.1	0.9(−1.4,3.5)	15.9 ± 4.8	17.3^∗^±4.5	1.3(−1.1,3.7)
	Muscle thickness (cm)	2.3 ± 0.3	2.5^∗^±0.3	0.2(0.1,0.4)	2.1 ± 0.3	2.2^∗^±0.3	0.1(0.02,0.2)	2.1 ± 0.3	2.3^∗^±0.3	0.1(0.1,0.2)
	Dm (mm)	6.1 ± 1.5	3.9^∗^±1.6	−2.1(−3.3,−0.9)	6.6 ± 1.6	5.8^∗^±1.9	−0.8(−1.6,0.1)	7.0 ± 2.4	5.9^∗^±1.9	−1.2(−2.4,0.01)
	Tc (ms)	31.9 ± 7.0	33.6 ± 7.3	1.7(−4.1,7.4)	30.9 ± 5.5	30.4 ± 3.6	−0.5(−5.2,4.2)	33.6 ± 7.9	31.8 ± 5.9	−1.8(−5.7,2.2)

*All data presented as mean ± SD; CI, 95% confidence limits; ^∗^, significant change over time (ANOVA), *P* < 0.*

**FIGURE 6:**
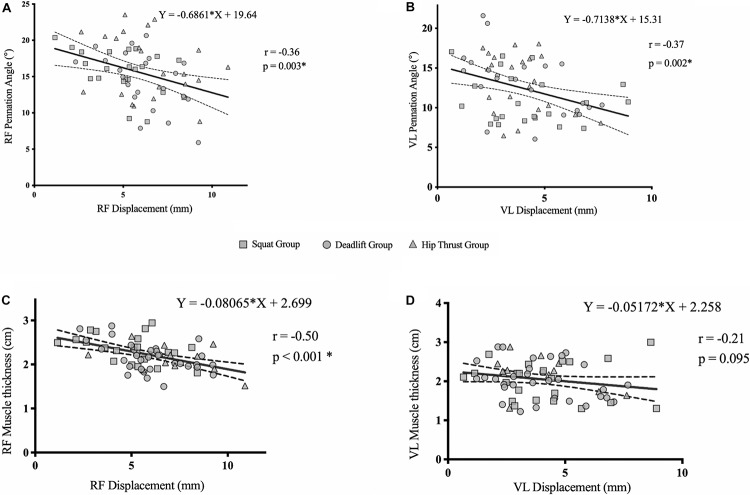
Pooled correlation analysis (*n* = 66) of pennation angle vs. radial muscle Dm in the RF **(A)**, VL **(B)**, muscle thickness vs. radial muscle belly Dm in the RF **(C)**, and VL **(D)**, of all exercise groups.

As no group interaction effects were seen in measures of muscle architecture and Dm, pooled correlational analysis was performed to investigate the relationship between muscle architecture and Dm; with pre- and post- measures of each variable (pennation angle, muscle thickness and Dm) being pooled together (*n* = 66). There were moderate negative relationships found between Dm and pennation angle in the RF (*r* = −0.36; *p* = 0.003); and the VL (*r* = −0.37; *p* = 0.002) (Figures 6A,B). Furthermore, pooled correlational analysis between muscle thickness and Dm revealed a moderate negative relationship in the RF (*r* = −0.50; *p* < 0.001); but not in the VL (*r* = −0.21; *p* = 0.095) (Figures 6C,D).

## Discussion

Following 3 distinct RT interventions, RF Dm was reduced alongside an increase in pennation angle and muscle thickness. Adaptations were not uniform across the knee extensors; as no between-group differences were seen in any measure of contractile properties or muscle architecture. However, muscle contractile properties and architectural adaptations were accompanied by increases in the maximal strength of all three exercises by each training group, indicating an improvement in overall lower limb strength and muscle function. Measures of RF and VL radial Dm were inversely associated with RF and VL pennation angle, respectively. However, only RF Dm was associated with muscle thickness, supporting the potential use of TMG to detect and measure muscle specific adaptations to RT.

To our knowledge this is the first study to employ TMG to assess muscle-specific adaptations in response to RT aimed at increasing lower limb strength. Previously, acute changes in Dm following exercise have been investigated and been shown to track exercise-induced fatigue, and decreased neuromuscular function (García-Manso et al., 2011, 2012; Hunter et al., 2012; Macgregor et al., 2016). Longitudinal alterations in Dm, like those of the present study, are less understood, likely owing to the inherent difficulty in assessing muscle stiffness *in vivo* (Macgregor et al., 2018). Nevertheless, TMG is capable of determining that more rapid Tc and lower Dm values are associated with greater exposure to strength and power training (Loturco et al., 2015; De Paula Simola et al., 2016; Šimunič et al., 2018); and that the increased muscle belly stiffness was contributory to improved force transmission (García-García et al., 2013; Hughes et al., 2015). Early work by Wilson et al. (1994) supports this by showing oscillation techniques to infer relationships between musculotendinous stiffness and isometric, concentric and eccentric force production; which was corroborated by later mechanomyography studies (Yoshitake et al., 2005, 2008). More recent support was shown by reduced lower limb muscles’ Dm seen following 8 weeks of plyometric training (Zubac and Simunic, 2017) and declined RF Dm observed following 3-weeks of strength focused training (Rusu et al., 2013). Both studies concluded that increased muscle stiffness (or muscle tone) contributed to improved muscle function and performance. Such findings support our current study as we demonstrated reductions in RF Dm were accompanied by improvements in 1RM strength in the BS, DL, and HT exercises.

Alterations in muscle thickness and pennation angle are known to occur as part of the hypertrophic response to RT, and are associated with increased muscular strength (Rutherford and Jones, 1992; Alegre et al., 2006; Strasser et al., 2013; Franchi et al., 2014), which is reflected in the findings of the current study. Along with the observed reduction in Dm, the observed increases in muscle architectural measures partially confirm our initial hypothesis of seeing alterations within the knee extensors. Previously, increases in pennation angle have been seen to contribute to increase PCSA of pennate muscle, thereby increasing its maximal force production capacity (Kawakami et al., 1995; Aagaard et al., 2001). Increases in pennation angle are thought to allow more contractile material to be packed into the same anatomical cross-sectional area (ACSA), increasing PCSA, thus allowing a greater number of fascicle-tendon attachments (Blazevich, 2006). The number of fascicle-tendon attachments directly contributes to a muscle’s maximal force generating capacity and therefore its overall strength. The significant increases in RF and VL muscle thickness seen in the present study, alongside increases in maximal strength are in agreement with current literature (Blazevich et al., 2003; Franchi et al., 2018). These aforementioned mechanisms responsible for improving maximal force transmission to pull direction, may, partly explain increased 1RM strength seen in the three exercise tests by all groups (Table 1). However, increased muscle thickness was not accompanied by an increase in pennation angle within the VL (Table 2). Potential explanations for this could be that increased VL muscle thickness was a result of an increased ACSA and not PCSA, which pennation angle has been previously associated (Aagaard et al., 2001). Additionally, architectural adaptations may not be uniform across quadriceps muscles (Mangine et al., 2018) and greater habitual usage of specific muscles may mean greater levels of hypertrophic response are required (Ema et al., 2013b). As only the mid-points of the RF and VL muscles were measured in the present study, it must be acknowledged that intra-muscular differences in architectural changes can occur (Ema et al., 2013a); owing to muscles’ region-specific functional roles (Watanabe et al., 2012). Future studies investigating muscle specific architectural adaptations in response to exercise should look to take measurements from multiple sites along a muscle’s length.

No statistically significant between-group differences were seen in architectural or contractile property adaptations (Figure 5); suggesting that training the three exercises had no differential effects upon measures in the RF or VL, which is at odds with our second hypothesis. Whilst the BS and DL have both shown high levels of VL and RF activation (Lee, 2015), quadriceps activation in the HT has not been as extensively investigated. Contreras et al. (2015) showed no difference between peak VL activation of the BS and HT in trained women, postulating that the quadriceps play a stability role in performing the HT movement despite the biomechanically distinct movement patterns of the respective exercises. As the RF has been shown to stabilize the pelvis during coordinated movements eliciting anterior and posterior tilting (Neumann, 2010; Lee, 2015), it may be possible that HT RT could have caused sufficient stimulus for RF adaptation to occur from this stability role; particularly in the untrained population of this study. However, as RF activation profiles and pelvic tilt kinematics in the HT have not been investigated to date, these are areas that require further investigation to confirm.

Physiological development of muscle tissue can be characterized by measures of muscle architecture and more recently by changes in muscle belly stiffness using elastography (Gennisson et al., 2010; Debernard et al., 2011; Eby et al., 2015; Lima et al., 2017). Debernard et al. (2011) observed across different ages groups that increases in fascicle angle were associated with increases in shear wave velocity (from which shear modulus was calculated) as a measure of muscle stiffness, or passive tension. These authors suggested that such information regarding contractile mechanics obtained from muscle belly stiffness assessments, may be useful when assessing alterations in muscle fascicle orientation. Whilst the aforementioned literature supports the association of muscle architecture and Dm parameters, it must be acknowledged that investigations into the measurement construct of techniques such as shear wave elastography, is ongoing (Taljanovic et al., 2017). The significant negative associations found within the VL and RF of the current study are in agreement (Figure 6), and suggests, that measurements of muscle belly Dm are reflective of increased pennation angle and muscle thickness; thus confirming our tertiary hypothesis. Mechanistically, angle of fascicle pennation will change as result of a change in muscle tension (Erskine et al., 2010). A change in muscle–tendon length, brought about by a change in joint angle, affects both the passive tension generated by the connective tissue (in parallel and in series) and the position of the contractile elements of the muscle. This, in turn, determines the level of tension that can be generated (Mohamed et al., 2002; Hunter et al., 2012). Pišot et al. (2008) found a similar relationship to our present study, between muscle thickness reductions and decreases in muscle belly stiffness. Reduced muscle belly stiffness was attributed to declined passive muscle tension, which accompanies muscle atrophy (Whitehead et al., 2001). Similarly, Šimunič et al. (2019) recently showed that changes in pennation angle and muscle thickness were significantly associated with changes in Dm following muscle atrophy, and actually preceded the associated measurable changes in muscle architecture. A potential limitation of the work by Šimunič et al. (2019) and the present study, is the absence of a control group. In the context of muscle adaptations, such data could add strength to the observed findings of the present study. This is an area future research should look to address. Additionally, future research should look to investigate the time course of adaptations and associations observed here; as only end-point measures were taken in the present study. The ability of Dm to reflect differing degrees of fascicle pennation may aid practitioners in monitoring, and implementing interventions alongside established physiological markers, perhaps following a RT program or within rehabilitation settings. As the present study was conducted with no-resistance trained individuals, future research should investigate the applicability of these findings in trained/athletic populations. TMG assessment of skeletal muscle contractile properties provides a unique insight into the rate of excitation-contraction coupling alterations in relation to architectural adaptations. Crucially, TMG may not only provide information on muscle architecture, but could also pose a more objective and simpler method of assessment; one that involves assessing skeletal muscle in an ‘active’ state as opposed to the resting requirements of ultrasonography.

## Conclusion

Tensiomyography detected a muscle specific training adaptation within the knee extensors, in response to 6-weeks of lower limb RT. Furthermore, measures of muscle belly Dm were associated with corresponding measures of muscle architecture, indicating that assessment of contractile properties can also provide information regarding muscle geometry that influences contractile mechanics. Whilst not only addressing a gap in the literature, concerning the longitudinal use of TMG, the findings of current study would be of benefit to practitioners and athletes in their efforts to investigate isolated muscle function. Applications of non-invasive contractile property assessments would provide objective insights into rehabilitation programs and the impact of specific interventions upon contractile mechanics and muscle adaptations within specific muscles. Furthermore, the objectivity and ability of TMG to assess contractile properties during an “active” contraction provides a unique insight into contractile mechanics. To further our understanding, relationships found in this study should be investigated in range of other muscles that play key roles in human movement. Additionally, similar RT adaptations should be studied in aging and female populations, as well as potential associations between such adaptations and measurable changes in performance outcomes.

## Data Availability Statement

All datasets generated for this study are included in the article/Supplementary Material.

## Ethics Statement

This study was carried out in accordance with the recommendations of the University of Stirling’s School of Sport Research Ethics Committee with written informed consent from all subjects obtained. All subjects gave written informed consent in accordance with the Declaration of Helsinki. The protocol was approved by the University of Stirling’s School of Sport Research Ethics Committee.

## Author Contributions

MW contributed to the study design, data collection, data analysis and interpretation, and writing of final manuscript. AR and SV contributed to the study design, ethics application, data collection, and preparation of final manuscript. AD-D contributed to the data collection, coaching during training sessions, and preparation of final manuscript. JD contributed to the ethics application, coaching during training sessions, and preparation of final manuscript. AH contributed to the study design, laboratory supervision, data analysis and interpretation, and preparation of final manuscript. DH  wrote the ethics application, contributed to the study design, supervision of study, data analysis and interpretation, and writing of final manuscript. LM contributed to the study design, data analysis and interpretation, study supervision, and writing of final manuscript.

## Conflict of Interest

The authors declare that the research was conducted in the absence of any commercial or financial relationships that could be construed as a potential conflict of interest.
