# Supplementary material for: Tensiomyography Derived Parameters Reflect Skeletal Muscle Architectural Adaptations Following 6-Weeks of Lower Body Resistance Training
Source: Front Physiol. 2019 Dec 10;10:1493. doi: 10.3389/fphys.2019.01493 (PMC6914863; doi:10.3389/fphys.2019.01493)
Supplement: Supplementary file 1 [file Data_Sheet_1.PDF]

Supplementary material

Images of exercise technique standards to which participants were coached to (referred to in line 287).

Back squat:

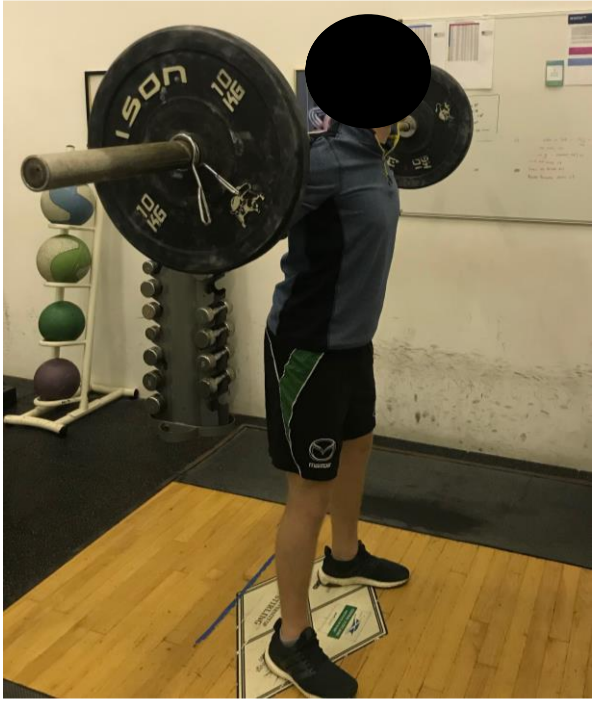

Start/finish position

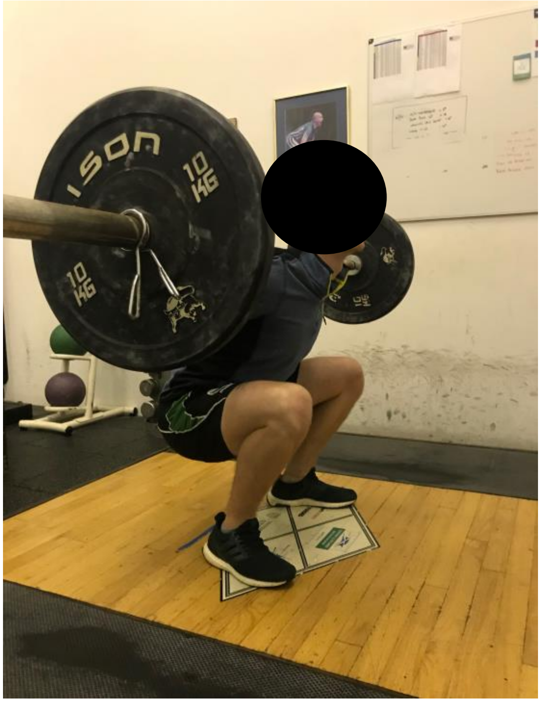

Bottom position

Deadlift:

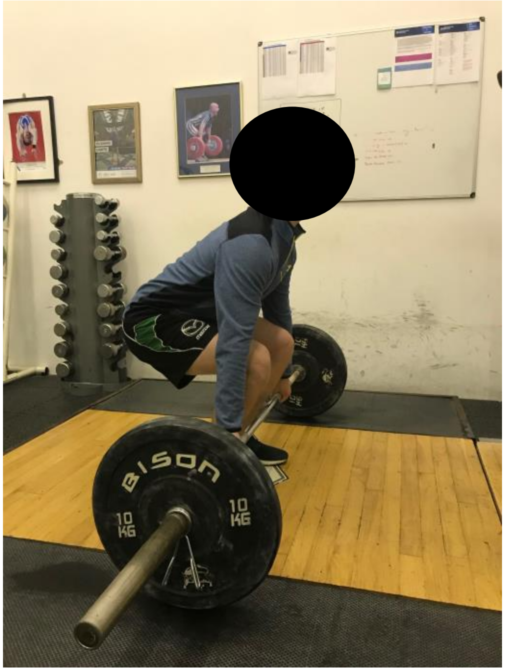

Start/finish position

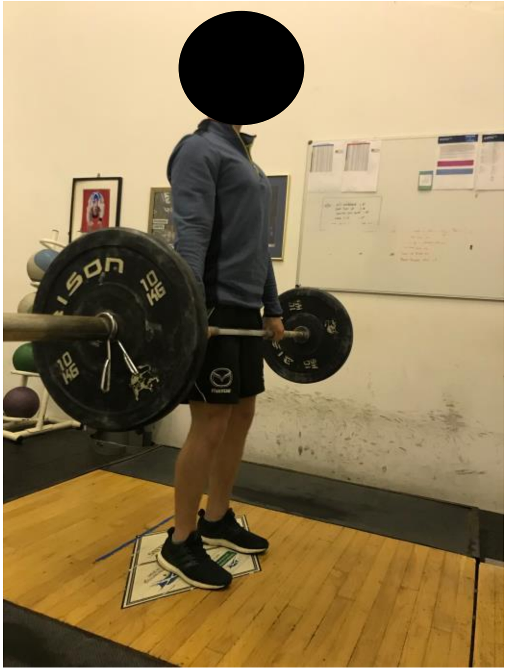

Top position

Hip Thrust:

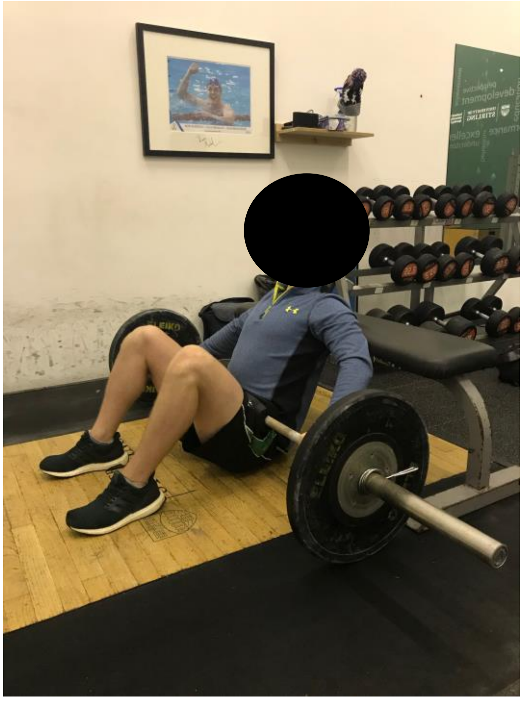

Start/finish position

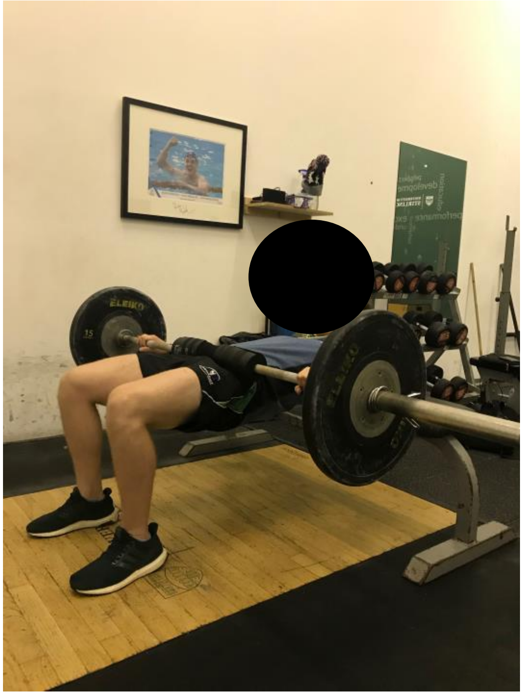

Top position
